# CT texture analysis: a potential tool for predicting the Fuhrman grade of clear-cell renal carcinoma

**DOI:** 10.1186/s40644-019-0195-7

**Published:** 2019-02-06

**Authors:** Zhan Feng, Qijun Shen, Ying Li, Zhengyu Hu

**Affiliations:** 1 Department of Radiology, First Affiliated Hospital of College of Medical Science, Zhejiang University, Hangzhou, 310003 Zhejiang China; 2 Department of Radiology, Hangzhou First People’s Hospital, Hangzhou, Zhejiang, 310003 China; 3 Department of Radiology, Second People’s Hospital of Yuhang District, Hangzhou, 310003 Zhejiang China

**Keywords:** Clear cell renal cell carcinoma, Computed tomography, Fuhrman grade, Heterogeneity, Texture analysis

## Abstract

**Background:**

The purpose of this study was to analyze the image heterogeneity of clear-cell renal-cell carcinoma (ccRCC) by computer tomography texture analysis and to provide new objective quantitative imaging parameters for the pre-operative prediction of Fuhrman-grade ccRCC.

**Methods:**

A retrospective analysis of 131 cases of ccRCCs was performed by manually depicting tumor areas. Then, histogram-based texture parameters were calculated. The texture-feature values between Fuhrman low- (Grade I-II) and high-grade (Grade III-IV) ccRCCs were compared by two independent sample t-tests (False Discovery Rate correction), and receiver operating characteristic curve (ROC) was used to evaluate the efficacy of using texture features to predict Fuhrman high- and low-grade ccRCCs.

**Results:**

There were no statistical differences for any texture parameters without filtering (*p* > 0.05). There was a statistically significant difference between the entropy (fine) of the corticomedullary phase and the entropy (fine and coarse) of the nephrographic phase after Laplace of Gaussian filtering. The area under the ROC of the entropy was between 0.74 and 0.83.

**Conclusions:**

Computer tomography texture features can predict the Fuhrman grading of ccRCC pre-operatively, with entropy being the most important imaging marker for clinical application.

## Background

Renal carcinoma is the most common adult malignant epithelial tumor of the kidney [1]. The most common pathological type is clear-cell renal cell carcinoma (ccRCC), which accounts for 60–85% of renal carcinoma [2]. Fuhrman grade is currently the most common classification method for ccRCC. It classifies renal carcinoma into four different pathological grades according to the size, shape, staining, and presence or absence of nucleoli of the nuclei of cancer cells. Among them, Grades I–II are low and Grades III–IV are high [3]. Current studies have shown that the grade of Fuhrman classification is closely related to the growth rate of tumors and the prognosis of patients [4–6]. High-grade tumors have higher invasive capacities, higher possibility of metastasis, and poor prognosis [7].

Fuhrman grade is an independent prognostic factor [8]. Currently Nephron-sparing surgery has been used more and more in the treatment of T1-stage ccRCC. But T1 tumors with a high Fuhrman grade had a higher malignant potential for subsequent tumor recurrence [9]. Therefore, pre-operative confirmation of the Fuhrman grading of ccRCC is critical for the selection of surgical options and prognosis.

Computer tomography (CT) texture analysis (TA) is a method used to quantitatively analyze spatial heterogeneity of lesions on CT images [10]. It is filtered by a Laplace of Gaussian (LoG) operation to reduce photon noise while highlighting the changes in the characteristics of the grayscale images [11, 12]. It has been applied to the identification of renal tumors [13], intraductal papillary mucinous neoplasm classification of the pancreas [14], and benign and malignant prediction of mediastinal lymph nodes [15].

This study aimed to explore the correlation between the texture features of ccRCC and Fuhrman grading by CTTA and to set up objective and effective quantitative parameters of imaging to evaluate the heterogeneity of ccRCC, and further help to predict the pre-operative Fuhrman grading and improve the prognosis of the patients.

## Methods

This retrospective study was approved by the Institutional Review Board of First Affiliated Hospital of College of Medical Science, Zhejiang University, and the requirement for informed consent was waived.

### Patients

The study retrospectively analyzed 131 patients with ccRCC from June 2013 to December 2017. The inclusion criteria were the following: (1) patients underwent pre-operative renal enhanced CT scans, including noncontrast phase, corticomedullary phase, and nephrographic phase; (2) the patients were scanned using the same scanning protocol on the same CT scaner; (3) the lesions showed at least seven slices on the CT axial images; (4) tumors were surgically removed or confirmed as ccRCC via percutaneous biopsy; (5) cases were not treated before CT scan. Exclusion criteria were as follows: (1) cases showing Fuhrman-grade ambiguity, such as undetermined between Grade II and III, and (2) CT images having obvious artifacts.

According to the Fuhrman classification, we divided the cases into a low-grade group (Grade I–II) and a high-grade group (Grade III–IV). There were 77 patients in the low-grade group, 59 males, and 16 females, aged 25–81 (53.82 ± 15.88) years; there were 54 high-grade patients, 41 males and 13 females, aged 48–77 (61.59 ± 11.52) years. All cases in this study were unilateral lesions.

### CT examination

All cases were scanned using a GE Lightspeed 64-row CT machine with a tube voltage of 120 kV and a tube current of 150–350 mA, and the non-ionic contrast agent (iodine content 300 mg/mL) was infused into the peripheral vein at an infusion rate of 3 mL/s and an infusion dose of 80–100 mL. The scan ranged from the adrenal gland to the inferior pole of the kidney with a thickness of 3 mm per layer. If the tumor had a large diameter, the scan was expanded to include the entire tumor. The corticomedullary phase was delayed by 30 s and the nephrographic phase was delayed by 90 s. We manually traced the region of interest (ROI) based on the boundary of the tumor on the CT images of the corticomedullary and nephrographic phases. The boundary of the lesion could not be accurately identified on the noncontrast phase images, which was not used in this study. We used two radiologists with 10 years of work experience to do ROI delineation, and two radiologists segmentation results were superimposed to generate the final ROI. Each ROI was validated by a senior radiologist with 20 years of experience. ROI sketching are completed in the self-compiled MATLAB (version R2013b, MathWorks, USA) program.

### Texture analysis

**Fig. 1:**
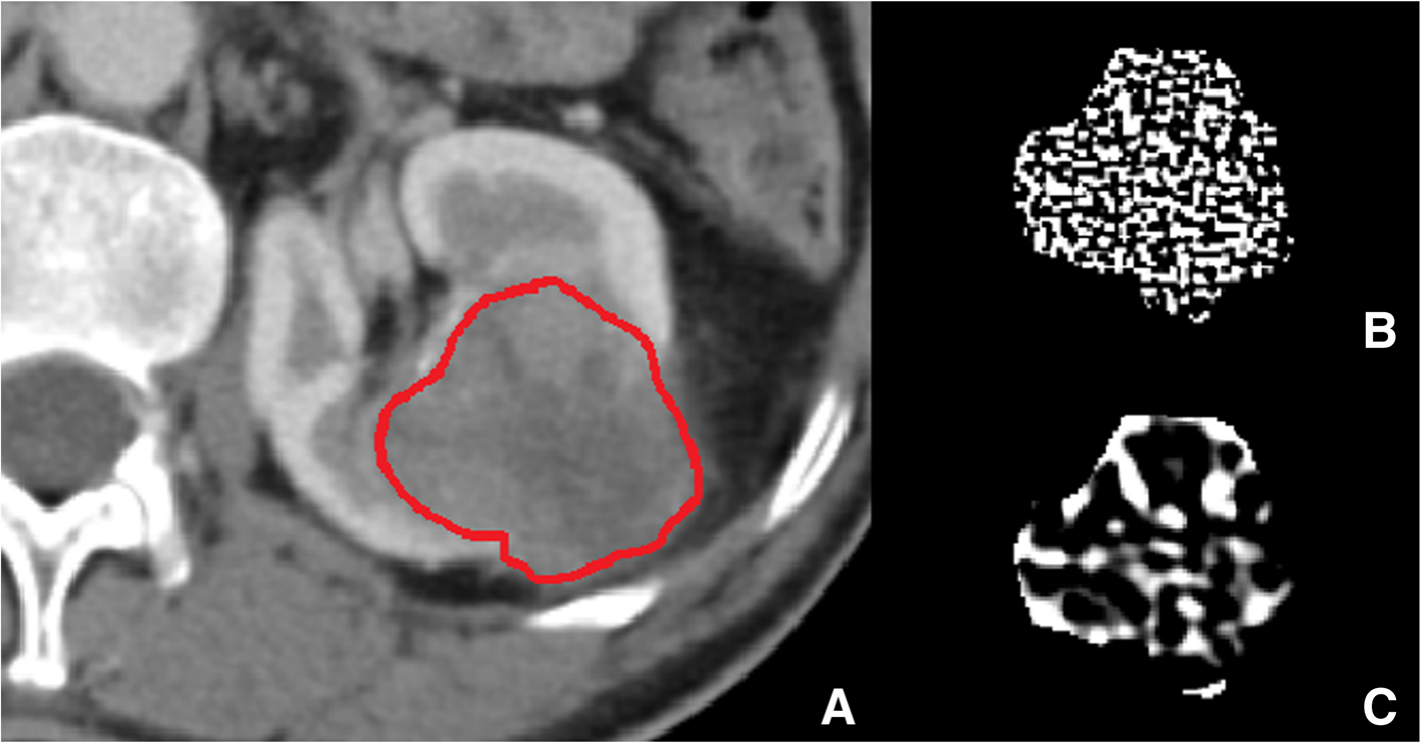
CT texture analysis to evaluate a clear-cell renal cell carcinoma(ccRCC). **a** Axial CT images of ccRCC texture feature before filtering, ROI was created within the confines of the tumor for texture analysis. **b** ccRCC texture feature after fine-scale filtering. **c** ccRCC texture feature after coarse-scale filtering

The data processing of texture analysis was also based on MATLAB, and the algorithm was basically consistent with the current published partial CT texture-analysis research [16–19]. The texture analysis included the following two processes: (1) filtering of the original images using LoG filtering. In this study, we used sigma (s) values of 1.0 (fine-scale filtration) and 2.5 (coarse-scale filtration), and the original unfiltered CT images were preserved (Fig.1). (2) Quantification of image texture. Currently, there are several texture parameters. In this study, only the most representative first-order histogram textures were calculated, including mean gray intensity, entropy, standard deviation, skewness, and kurtosis. The mean gray intensity is the average of the pixel intensities, and the entropy is the amount of information contained in the aggregated features of the grayscale distribution in the images, reflecting the complexity of the images. The standard deviation is the degree of dispersion of individual pixel values in the grayscale distribution of the images. Skewness is a quantitative indicator of histogram asymmetry, and kurtosis is a quantitative indicator of histogram spikes.

### Statistical analysis

This study used version 3.3.2 of R software (http://www.R-project.org) for statistical analysis of the data. The texture parameters of the Fuhrman low−/high-grade groups were compared using a two-sample t-test. In order to avoid false positives after multiple comparisons, we also performed multiple comparison corrections based on False Discovery Rate (FDR), with a statistical difference of *p* < 0.05. Subsequently, the receiver operating characteristic (ROC) curve analysis was performed on the statistically significant texture parameters, and the area under the ROC curve (AUC) and sensitivity and specificity values were calculated to evaluate the effectiveness of each parameter in distinguishing the Fuhrman grading.

## Results

Table 1 shows that no texture parameters of the two groups were statistically different (*p* > 0.05) without the addition of filtering. The mean gray intensity and skewness showed no significant differences after LoG filtering (*p* > 0.05). There was a statistically significant difference in standard deviation of the corticomedullary phase (coarse filtration) (*p* < 0.05), and there were significant differences in kurtosis (coarse filtration) of the corticomedullary and nephrographic phases (p < 0.05). There were statistically significant differences in all entropy after LoG filtering (*p* < 0.01). However, entropy (fine filtration) of the corticomedullary phase and entropy (fine and coarse filtration) of the nephrographic phase showed significant statistical differences after FDR corrections.

**Table 1:** CCTA Parameters of low and high grade clear cell renal cell carcinomas

Parameter	Grade I–II	Grade III-IV	*p* value	Adjusted *p* value
Corticomedullary phase				
Mean grey-level intensity				
No filtration	93.95 ± 36.49	81.99 ± 30.21	0.14	0.29
Fine filtration	−0.43 ± 0.23	− 0.35 ± 0.31	0.19	0.37
Coarse filtration	−0.59 ± 0.37	−0.47 ± 0.34	0.30	0.43
Standard deviation				
No filtration	39.17 ± 18.27	32.18 ± 15.78	0.26	0.37
Fine filtration	6.29 ± 2.45	4.89 ± 3.11	0.10	0.26
Coarse filtration	2.43 ± 1.29	1.59 ± 1.03	0.04*	0.17
Kurtosis				
No filtration	5.43 ± 8.31	10.21 ± 19.33	0.18	0.32
Fine filtration	5.29 ± 3.99	8.12 ± 4.21	0.30	0.37
Coarse filtration	4.89 ± 4.72	10.24 ± 15.19	0.04*	0.17
Skewness				
No filtration	−0.11 ± 0.58	− 0.06 ± 0.64	0.87	0.90
Fine filtration	−0.26 ± 0.61	−0.28 ± 0.69	0.93	0.94
Coarse filtration	−0.56 ± 0.54	−0.62 ± 0.60	0.69	0.74
Entropy				
No filtration	6.92 ± 0.43	6.76 ± 0.60	0.18	0.32
Fine filtration	4.73 ± 0.23	4.53 ± 0.29	0.003*	0.04*
Coarse filtration	3.10 ± 0.40	2.80 ± 0.52	0.007*	0.05
Nephrographic phase				
Mean grey-level intensity				
No filtration	84.33 ± 23.53	75.55 ± 18.81	0.09	0.26
Fine filtration	−0.28 ± 0.21	−0.20 ± 0.19	0.27	0.37
Coarse filtration	−0.40 ± 0.35	−0.32 ± 0.28	0.36	0.43
Standard deviation				
No filtration	24.97 ± 17.94	19.36 ± 13.85	0.29	0.38
Fine filtration	4.82 ± 2.54	3.39 ± 2.34	0.13	0.29
Coarse filtration	1.72 ± 1.40	1.31 ± 0.69	0.08	0.26
Kurtosis				
No filtration	6.25 ± 8.66	4.97 ± 4.41	0.12	0.29
Fine filtration	5.14 ± 7.53	9.50 ± 11.83	0.18	0.33
Coarse filtration	5.66 ± 4.67	7.48 ± 9.72	0.02*	0.15
Skewness				
No filtration	−0.46 ± 1.26	0.09 ± 1.88	0.13	0.30
Fine filtration	−0.22 ± 0.73	−0.94 ± 2.40	0.19	0.32
Coarse filtration	−0.56 ± 0.81	−0.93 ± 1.60	0.20	0.33
Entropy				
No filtration	6.52 ± 0.36	6.44 ± 0.43	0.39	0.44
Fine filtration	4.60 ± 0.22	4.29 ± 0.29	0.006*	0.04*
Coarse filtration	2.76 ± 0.32	2.37 ± 0.21	0.001*	0.03*

*P < 0.05

**Fig. 2:**
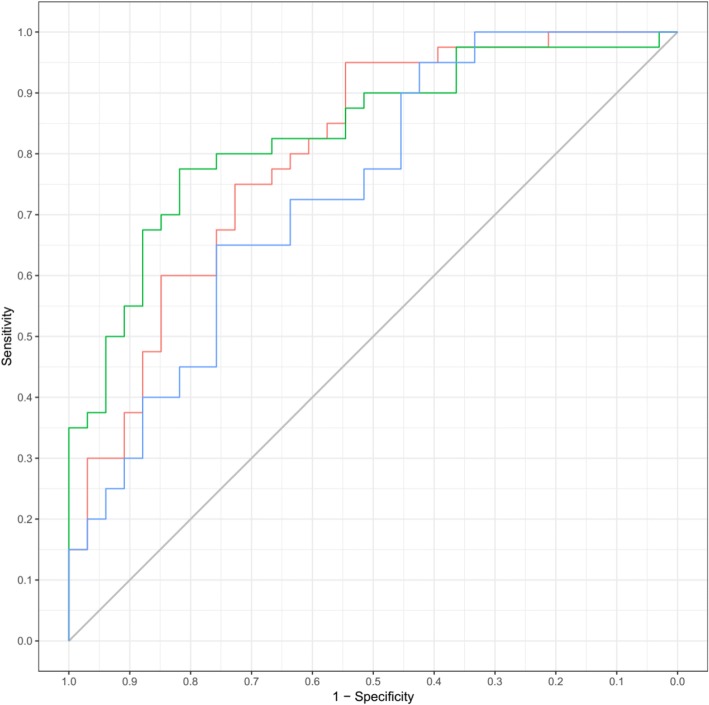
ROC analysis of entropy values. The corticomedullary phase entropy (fine filtration) is blue. The entropy (fine filtration) of the nephrographic phase is red. The nephrographic phase entropy (coarse filtration) is green

Table 2 lists the AUC, the optimal threshold, and the corresponding accuracy after FDR correction of the entropy-predicted Fuhrman low/high grades. The corticomedullary phase entropy (fine filtration) had an AUC of 0.74, sensitivity of 0.76, specificity of 0.65 and cutoff value of 4.66. The entropy (fine filtration) of the nephrographic phase had an AUC of 0.80, sensitivity of 0.95, specificity of 0.54 and cutoff value of 4.27. The nephrographic phase entropy (coarse filtration) had an AUC of 0.83, sensitivity of 0.82, specificity of 0.77 and cutoff value of 2.55(Fig.2).

**Table 2:** Accuracy of CTTA predictive performance under different filters

Parameter	AUC	Sensitivity	Specificity	Cutoff
Corticomedullary phase				
Entropy(fine)	0.74	0.76	0.65	4.66
Nephrographic phase				
Entropy(fine)	0.80	0.95	0.54	4.27
Entropy(Coarse)	0.83	0.82	0.77	2.55

## Discussion

There is an important relationship between the Fuhrman classification of ccRCC and prognosis, so several non-invasive methods have been used to predict the Fuhrman grading of ccRCC. MR(Magnetic Resonance)has functional imaging studies based on diffusion and perfusion [20, 21], but MR is costly, which leads to lower popularity. Wang et al. used the RENAL nephrometry score based on CT images for the prediction [22]. There have also been a large number of CT-based semi-quantitative and quantitative studies [23, 24], which indicates that CT is a convenient and effective method for predicting the Fuhrman classification of ccRCC. In this study, we further explored the CT data. After applying the LoG filter to pre-process the images, entropy was found to be a very important factor in predicting the texture parameters of Fuhrman grading.

The results suggest that there was no statistically significant difference in any texture parameters of the unfiltered images. Previously, Huhdanpaa et al. also performed Fuhrman-grade prediction based on the histogram parameters of ordinary CT images. The results show that only the inter-quartile range of the nephrographic phase has significant statistical significance. There was no statistical difference in mean, standard deviation, skewness, and kurtosis [25], which was consistent with our unfiltered results. Further, we carried out LoG filtering and found that some texture parameters showed significant differences after LoG filtering. LoG filtering is an advanced image-filtering method that combines Laplacian filtering and Gaussian filtering. Laplacian filtering can highlight the grayscale mutation region in the image and enhance the grayscale contrast. Gaussian operators can suppress the noise brought about by the Laplacian operator [11, 26]. The low filter value corresponds to the fine texture features and the high filter value corresponds to the coarse texture features. Meghan and others also found that only LoG filtered texture parameters were significantly correlated with cirrhosis grade [27], and our results once again confirm that LoG filtering can improve the ability to detect disease heterogeneity.

Heterogeneity is an important feature of malignant tumors and is closely related to the adverse biological processes of tumors. CTTA is a technique for effectively assessing tumor heterogeneity [12, 28]. Zhu et al. retrospectively evaluated 255 cases of ccRCC and found that low enhancement of medullary tumors was an independent factor in predicting high-grade tumors, but their experiments required higher ROI and extensive experience of the physician in measurements to avoid areas of obvious necrosis, large blood vessels, and calcification. In addition, they only selected one slice of the tumor imaging, but not the entire tumor [29]. The method of Zhu et al. relied too much on personal experience, and the consistency between the measures was difficult to guarantee. Pichler et al. believed necrosis to be an independent prognostic indicator of ccRCC, so that avoiding necrotic regions almost completely ignored the heterogeneity of ccRCC, which was an important feature of tumors [30]. Hebert et al. performed Fuhrman-grade analysis based on single slices and the entire lesion, and they found that the pathological grade was not associated with the enhancement parameters of a single slice, while the enhancement parameters of the volume measurement were related to the grade [31]. Choosing the suitable slice is important when only analyzing one slice of image. However, the result of a single slice cannot fully reflect the heterogeneity of the entire tumor. Therefore, this study used volumetric measurements, which were relatively cumbersome, but the assessment of heterogeneity was very important.

Our study aimed to reveal a group of common first-order parameters to reflect tumor heterogeneity, including standard deviation, mean gray intensity, skewness, kurtosis, and entropy. It is worth mentioning that we have introduced entropy, which is an abstract quantitative indicator of texture irregularity and chaos, reflecting the degree of disorder of the images [11]. All of these parameters can reflect the overall texture features of the tumor to some extent, and are used to objectively and quantitatively analyze the heterogeneity of ccRCC. The final result verifies our assumption that entropy is indeed an independent and excellent texture parameter. However, counterintuitively, the average entropy of low-grade tumors is higher than that of high-grade tumors; that is, the higher heterogeneity was found in low-grade tumors. However, this result is consistent with the results of Heidi et al. and Kousei et al [24, 31]. We speculate that it may be due to the fact that the micro-vessels of the lower-grade tumors are more abundant and the degree of intra-tumor intensification is higher. In addition, the concept of entropy is relatively abstract. The larger entropy value indicates a more random density distribution in the lesions, and the smaller entropy value indicates a relatively uniform density distribution. High-grade tumors, due to their relatively large liquefaction necrosis range, will result in reduced entropy.

In this study, there was a statistically significant difference in the standard deviation (coarse filtration) of the corticomedullary phase and the kurtosis (coarse filtration) of the corticomedullary and nephrographic phases before FDR correction, but they were not corrected by multiple comparisons. Chalkidou pointed out that there were serious false positives in the current CCTA studies [32]. Many studies have not performed multiple comparison corrections, resulting in poor reproducibility, and, accordingly, the results cannot be reproduced. In order to reduce the typeIerror, the study performed a strict FDR correction on the results. Surprisingly, entropy is statistically significant even after FDR correction, and in subsequent ROC curve analysis the entropy also showed a good degree of discrimination in tumor grades. The entropy calculation based on ordinary CT images is simple and convenient, and can objectively quantify the heterogeneity of tumors, and has the prospect of clinical transformation.

The limitations of this study were as follows. (1) This study was a single-center retrospective study with no external data validation. (2) The sample size was still relatively small. In fact, the number of low-grade Fuhrman tumors is very large, while the number of high-grade tumors is relatively small, and the number of cases between groups is easily imbalanced. (3) Since the tumor boundary is manually drawn, the interference of the volume effect cannot be completely avoided. (4) The WHO/International Society of Urologic Pathology grading system will replace Fuhrman grading [33]. In the future, we plan to collect cases based on new grading.

## Conclusions

This results of this study show that LoG filtering can reduce image noise while highlighting the degree of unevenness inside a tumor, which is a very effective image pre-processing method. The entropy value can objectively quantify tumor heterogeneity, and the calculation is simple and convenient. It can be directly applied to conventional CT to assist clinicians in predicting Fuhrman grade.

## Abbreviations

AUC: The area under the curve
ccRCC: Clear-cell renal-cell carcinoma
CT: Computed tomography
FDR: False Discovery Rate
LoG: Laplace of Gaussian
MR: Magnetic Resonance
ROC: Receiver operating characteristic
ROI: Region of interest
TA: Texture analysis
